# Improving the Care and Treatment of Monkeypox Patients in Low-Resource Settings: Applying Evidence from Contemporary Biomedical and Smallpox Biodefense Research

**DOI:** 10.3390/v9120380

**Published:** 2017-12-12

**Authors:** Mary G. Reynolds, Andrea M. McCollum, Beatrice Nguete, Robert Shongo Lushima, Brett W. Petersen

**Affiliations:** 1 US Centers for Disease Control and Prevention, Poxvirus and Rabies Branch, Atlanta, GA 30329, USA; azv4@cdc.gov (A.M.M.); ige3@cdc.gov (B.W.P.); 2 Kinshasa School of Public Health, Kinshasa 11850, Congo; beatanguete13@gmail.com; 3 Ministry of Health, Kinshasa 11842, Congo; robert_shongo@yahoo.fr

**Keywords:** monkeypox, *Orthopoxvirus*, standard of care, animal model, clinical syndrome

## Abstract

Monkeypox is a smallpox-like illness that can be accompanied by a range of significant medical complications. To date there are no standard or optimized guidelines for the clinical management of monkeypox (MPX) patients, particularly in low-resource settings. Consequently, patients can experience protracted illness and poor outcomes. Improving care necessitates developing a better understanding of the range of clinical manifestations—including complications and sequelae—as well as of features of illness that may be predictive of illness severity and poor outcomes. Experimental and natural infection of non-human primates with monkeypox virus can inform the approach to improving patient care, and may suggest options for pharmaceutical intervention. These studies have traditionally been performed to address the threat of smallpox bioterrorism and were designed with the intent of using MPX as a disease surrogate for smallpox. In many cases this necessitated employing high-dose, inhalational or intravenous challenge to recapitulate the severe manifestations of illness seen with smallpox. Overall, these data—and data from biomedical research involving burns, superficial wounds, herpes, eczema vaccinatum, and so forth—suggest that MPX patients could benefit from clinical support to mitigate the consequences of compromised skin and mucosa. This should include prevention and treatment of secondary bacterial infections (and other complications), ensuring adequate hydration and nutrition, and protecting vulnerable anatomical locations such as the eyes and genitals. A standard of care that considers these factors should be developed and assessed in different settings, using clinical metrics specific for MPX alongside consideration of antiviral therapies.

## 1. Introduction

Monkeypox (MPX) is a zoonosis afflicting people who live in communities in which contact with sylvatic animals is commonplace [1,2]. The range of animal species known to be susceptible to monkeypox virus (MPXV) infection includes multiple species that are utilized as priority or supplemental protein sources in rural, forested communities of the Democratic Republic of the Congo (DRC), where human monkeypox cases occur most frequently [3,4,5].

While most cases are reported from DRC, MPX occurs sporadically throughout heavily forested, and typically impoverished, regions of rural West and Central Africa, including Cameroon, Central African Republic, Republic of the Congo, and Sierra Leone [6,7,8,9]. The virus that causes MPX belongs to the genus *Orthopoxvirus*, alongside *Variola virus*, the causative agent of smallpox. There are two principal genetic clades of MPXV, one of which occurs in Central Africa, the other in West Africa [10,11]. Viruses in the West African genetic clade are thought to be less virulent, but relatively few instances of human infection with the West African variant of MPXV have been documented [12,13]. The fatality rate for MPX among non-vaccinated persons infected with the Central African variant of the virus has been estimated at 11–15% [14], which is slightly less than the lower range observed among persons with discrete, ordinary smallpox. 

**Figure 1:**
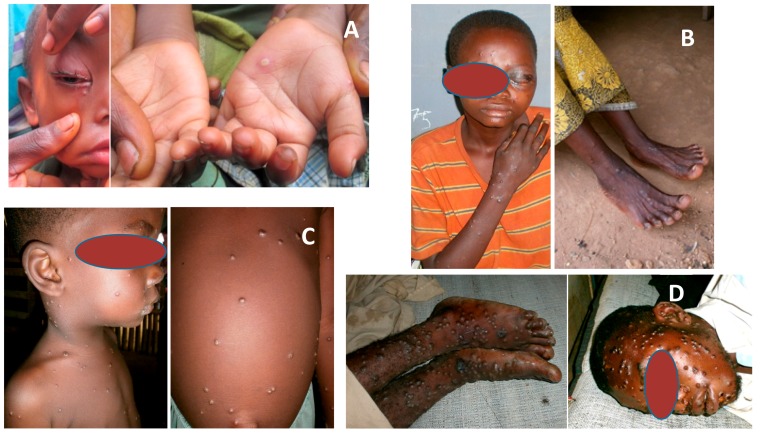
Spectrum of rash burden experienced by different individuals with acute monkeypox, Democratic Republic of the Congo. Lesion counts are based on whole-body estimates performed by trained health care personnel. (**A**) “benign”, 5–25 lesions (plus ocular involvement); (**B**) “moderee”, 26–100 lesions [plus ocular involvement]; (**C**) “grave”, 101–250 lesions (plus lymphadenopathy); (**D**) “plus grave”, >250 lesions. (Photo credits: (**A**) Jacque Katomba; (**B**,**D**) Gregoire Boketsu; (**C**) Toutou Likafi).

Monkeypox, like smallpox, begins with a brief (2–3 day) febrile prodrome period prior to the appearance of enanthem and then exanthema, the latter with centrifugal distribution. The total lesion burden at the apex of rash can be quite high (>500 lesions) or relatively slight (<25) (Figure 1). Monkeypox chiefly occurs in communities where there is often a high background prevalence of malnutrition, parasitic infections, and other significant heath-compromising conditions, any of which could impact the prognosis of a patient with MPX. Patients are subject to a range of complications that can include secondary infection of the integument, bronchopneumonia, sepsis, encephalitis, and infection of the cornea with ensuing loss of vision [12,14,15,16,17]. These were also, for the most part, notable complications of infection with variola virus. However, while these illnesses are similar in many ways, they are not equivalent. Several features delineate the two illnesses: (i) the prominence of lymphadenopathy for monkeypox; (ii) the greater significance of parenteral modes of infection for MPX (e.g., transdermal, mucosal; presumably stemming from contact with MPXV-infected wildlife and wildlife carcasses); and (iii) fivefold higher efficiency of inter-human transmission for smallpox [18].

A significant challenge facing health care providers who work in rural DRC and other resource-poor settings is how to provide an appropriate standard of care for patients with MPX when evidence-based treatment recommendations, and even the most basic supportive therapies, are lacking. The contemporary clinical picture of MPX in rural Africa is not understood with sufficient precision despite the fact that the disease is extant and the virus that causes it is actively circulating in a number of countries [4,7,8,19]. For example, there is a lack of understanding about the most common and most significant complications of illness, general or age-specific mortality rates, or rates of sequelae. Our current understanding rests largely on case series and individual case reports. These suggest that in the aftermath of smallpox eradication the loss of vaccine-derived immunity, since the eradication of smallpox has engendered fresh opportunities for MPX acquisition, but shortcomings in options for treatment have left those afflicted with little by way of standardized medical support aside from basic supportive care [20]. 

Biomedical advances from disease mitigation and treatment studies for other illnesses affecting the skin and airway (e.g., varicella, influenza) may, in some instances, also apply to MPX. Another largely untapped potential source of evidence to inform a general approach to care may be derived from recent investigations (performed during the last decade and a half) which have sought to characterize the pathophysiology and course of infection of smallpox, using MPXV as a surrogate. These studies were undertaken for biodefense purposes (reviewed in [21], largely to assess the adequacy of animal model systems for use in efficacy testing of smallpox medical countermeasures, or to otherwise generate observations that could be extrapolated to smallpox [22,23,24]. To date little attempt has been made to use the evidence generated from these studies, or those from immunologic studies or outbreak investigations, to attempt improvements for the clinical management of MPX in humans. Of particular importance are insights that could shed light on ways to optimize care in low-resource settings, where both the cost of care and the availability of basic pharmaceuticals and other supplies are limited. 

Here we summarize relevant evidence from contemporary biomedical and biodefense research and offer perspectives on approaching the clinical care of monkeypox patients. Further, we highlight several gaps in our current understanding of orthopoxvirus-associated disease in humans and suggest additional investigations. 

## 2. Skin

The hallmark feature of monkeypox is disseminated vesiculo-pustular rash. In instances of severe illnesses with high rash burden, this alone can create a substantial vulnerability for an infected individual. Recent MPX disease surveillance data collected from Tshuapa province, DRC, show that ~50% of monkeypox patients have extensive rash burdens (>100 lesions, personal communication, Robert Shongo, Ministry of Health, DRC) and most exhibited oral lesions, suggesting that many patients will have extensive injury to the skin and to mucosal surfaces. Histopathologic analysis of the earliest stages of lesion development in humans (papular, pre-vesicular) reveals epidermal necrosis at the center of individual lesions concurrent with nascent extension into the superficial layers of the dermis [25]. As observed in MPXV-infected non-human primates (NHPs), lesion pathology intensifies as pustules form, with progressive ulceration, necrosis and epithelial hyperplasia. Edema is prominent at the margins of necrotic areas and clefts develop in interstitial spaces between cells where fluid and cellular debris accumulate. Later, at the apex of lesion evolution, inflammation and necrosis of the superficial dermis predominates and destruction of sebaceous glands and follicles is evident [23]. Together these attributes lead to characterization of the affected areas as “partial thickness wounds” [26,27]. Injury of this extent points to the need for active prevention of secondary bacterial infection and possible cellulitis, which has been observed in the context of other Orthopoxvius-associated diseases [28,29]. The creation of a clean, moist microenvironment has been demonstrated to promote re-epithelialization of skin eroded at herpes simplex lesion sites and due to other severe dermatologic conditions (also “partial thickness wounds”). Interventional studies demonstrated that the use of moist occlusive therapies successfully promoted re-epithelialization and healing at herpes lesions sites [27,30,31]. While it may be impractical to attempt to apply whole-body occlusive dressings, the judicious use of moist occlusive dressings at particular locations—such as MPXV inoculation sites (e.g., animal bite locations) or sites with dense constellations or coalescent lesions—might prove to be of value to a patient.

Dermal healing is generally considered to progress through three discrete phases: inflammation, proliferation, and remodeling. Inflammation is prominent in the immediate aftermath of the wound but resolves as lesions progress toward desquamation. In general, slow- or non-healing wounds arrest at the inflammatory phase. In individuals suffering from MPX who have very high rash burdens (>250 lesions), interleukin-10, an anti-inflammatory, has been reported to be markedly elevated [32]. Yet when murine “full-thickness” wound explants are exposed to very high levels of exogenous, parapoxvirus-encoded IL-10, instead of dampening inflammation (thereby promoting healing, which is consistently observed with lower doses), the opposite occurs [33]. In NHPs experimentally infected with MPXV, IL-10 expression appears after the peak of illness severity, roughly coincident with the beginning of weight gain and recovery. Whether the level of endogenous stimulation of IL-10 in MPX patients with high rash burden potentiates or dampens inflammation, and how this might impact skin regeneration, is an open question. Also open to question is how an individual’s constituent microflora affects their epidermal healing capacity. Recent studies have suggested that a patient’s resident microbiome can impact healing, with relatively diverse communities of colonizing bacterial capable of inhibiting healing at mucosal surfaces owing to the release of soluble products that disrupt epithelial integrity [33,34]. 

Risks for secondary infection at sites of compromised skin, or at breaches on mucosal surfaces, have not been the subject of focused investigations, but the possible contribution of superinfections to the development of cellulitis or sepsis suggests that this should be a priority area for active research. Bacterial superinfection is hypothesized to contribute to scarring [35]. Dermal scarring attributable to the accumulation of granulation in tissues following inappropriate treatments, scratching or secondary infections is largely preventable through education of providers and behavioral modification by patients. Facial scaring, on the other hand, may be less avoidable if there is extensive destruction of sebaceous glands (which occur in abundance on the face) during illness [35,36]. The use of moist, occlusive dressings could be contemplated for patients with extensive facial coverage of rash lesions. 

## 3. Eyes

One of the most significant sequelae of monkeypox infection is corneal scarring and concomitant loss of vision [17]. Nearly 25% (68/294) of confirmed MPXV cases identified in the Tshuapa province of DRC, 2010–2013, reported “conjunctivitis” as a symptom of infection (personal communication, Robert Shongo, Ministry of Health, DRC). The proportion of these cases with uncomplicated eyelid involvement (blepharoconjunctivitis), versus those with keratitis or ulcerations of the cornea, is currently unknown. Prior studies suggest that infection of the cornea may be a relatively uncommon complication of MPX [16], but can have lifelong impacts. Further classification of the proportion of MPX patients having ocular complications who ultimately develop visual deficits would be useful. Ocular complications are not unique to MPX. They were observed in 5–9% of infections with variola virus [37]. In the decades prior to eradication, steps were often taken to protect the vision of patients at risk for blindness by applying lubricants to the eyes and providing the patient with vitamin supplementation [37]. This was perceived to be particularly important as a means to stave off secondary bacterial infections of the cornea, which tended to occur later during the course of illness. Bacterial superinfection of smallpox-induced corneal ulcerations was often associated with catastrophic damage to the eye (perforation, anterior staphyloma, phthisis bulbi) [37]. Detailed studies of MPX-induced loss of vision have not been performed.

Ocular infections with orthopoxviruses have been recently reported in the United States stemming from inadvertent implantation of vaccinia virus into the eye [38,39,40]. In many of these instances, topical application of liquid trifluridine has been employed to hasten resolution of symptoms and to prevent long-term damage from scarring. Trifluridine is considered to be the preferred treatment regimen for ocular vaccinia, but topical or oral antibiotics have also been used in combination either to treat bacterial superinfection or as prophylactic therapy [37,40,41]. Recrudescent corneal erosion from cowpox virus infection was reported in a patient nine months after the initial lesion had apparently healed (i.e., absence of culturable virus). The use of steroid drops to control inflammation is thought to have contributed to virus persistence and prolonged corneal damage in this patient [28], who ultimately received successive corneal transplants. In prior instances during which MPX patients with ocular complications were followed over successive years, persistent pain, corneal opacity, scar tissue, and long-term loss of vision were observed (personal communication, A. McCollum, CDC). For monkeypox, the potential benefits of relatively simple therapies for ocular complications, such as enhanced lubrication (used during the smallpox era) or topical antibiotics, could be considered. However, as with ocular vaccinia, specific, early therapy to promote virus elimination may be warranted in MPX patients in order to prevent long-term vision deficits. 

## 4. Systemic Illness

The route by which humans become infected with MPXV is thought to influence both the severity and the manifestations of MPX illness [40,42]. Infection can occur parenterally (e.g., animal bites, scratches or other breaks in the skin), via mucosal surfaces (eye, mouth), or through respiratory routes. Observations collected from patients infected with a West African genetic variant of MPXV during an outbreak in the United States demonstrated that parenteral exposures tended to be associated with more profound systemic illness, a greater likelihood of the patients’ experiencing nausea and vomiting, and a lesser probability of febrile prodrome, as parenterally exposed persons typically displayed an early inoculation lesion [42]. Mucosal infections have been less well described, but anecdotes emerging from sporadic case reports in Central Africa suggest that severe—sometimes fatal—mucocutaneous infections have ensued from ingestion of wildlife harboring the virus (personal communication, C. Moses, International Conservation Education Fund). While such reports remain anecdotal, their frequency and apparent clinical significance suggest that this phenomenon merits further investigation. 

Though administered at doses higher than that anticipated to be necessary to achieve infection via respiratory routes in humans, intratracheal, aerosol challenge of non-human primates with MPXV (~3.4–8.7 × 10^6^ plaque forming units) resulted, in one study, in the animals developing ulcerative stomatitis and necrotizing lesions distributed along their upper gastrointestinal tract [43]. The animals also demonstrated aberrant blood protein levels—hypoproteinemia and hypoalbuminemia—several days after the onset of inappetance, fever and rash. This finding was attributed in part to a deteriorating nutritional status owing to the animals’ apparent discomfort and aversion to eating and drinking. 

Similar observations of post-infection swelling of the face and throat were made among NHPs infected in laboratory settings [23], and among chimpanzees with naturally-acquired infection. The latter was observed during an outbreak that occurred at the Mefou Preserve in Cameroon, where affected animals were observed early during illness to have poor appetite and labored breathing concomitant with prominent neck and facial swelling (CDC personal communication). 

Hypoalbuminemia and low hematocrit, suggestive of malnutrition, was observed in patients who were hospitalized with monkeypox during the US outbreak in 2003 [12]. This was most apparent in those who had relatively more severe manifestations of infection (defined as three or more aberrant clinical chemistry values or duration of hospitalization >48 h). Of note, factors possibly contributing to diminished appetite—the appearance of mouth and throat sores, nausea and vomiting, cervical lymphadenopathy—occurred early during illness (the first six days after illness onset). 

Taken together, these observations suggest that some aspects of monkeypox-associated morbidity attributable to nutritional deficits and fluid compromise have the potential to be mitigated by nutritional supplementation and fluid resuscitation. This and adequate attention to controlling pain could help ensure that patients sustain the energetic requirements needed for dermal healing, and to avoid complications. Additionally, addressing inflammation (reducing swelling of lymph nodes in the head, throat, and neck) might serve to increase a patient’s willingness to accept food and water. However, the benefits of anti-inflammatories relative to the potential harm imposed through immune suppression should be carefully balanced.

## 5. Bronchopneumonia

A notable but poorly characterized complication of MPX—and before that, smallpox—is bronchopneumonia. The respiratory challenge of NHPs across a range of infectious doses has been reproducibly shown to result in the development of focal necrosis of lung tissues, diffuse pulmonary consolidation, and fulminant bronchopneumonia. In several studies, the intratracheal deposition of virus-containing aerosols led to significant respiratory distress and death (or euthanasia owing to moribund status) in a high proportion of animals [22,43,44]. The late onset of *Klebsiella pneumonia* was noted in one animal that succumbed to illness [43], but secondary bacterial infections were not generally noted in animals that died. 

The contribution of secondary airway infections in real-world clinical settings remains ill-defined. The frequent presence of “cocci” in pulmonary exudates of patients who died from smallpox-associated pneumonia led many to hypothesize that bacterial superinfection was an important cofactor in smallpox deaths, particularly among deaths that occurred late during illness [45]. Alternatively, animal studies tend to support the suggestion that bacterial superinfection can exacerbate pulmonary manifestations of illness, and bias toward severe outcomes, but that they are not required to bring about death, at least in instances of direct respiratory challenge. 

Infection with influenza virus has been shown to predispose individuals to secondary infection with common upper respiratory bacterial commensals [46]. Several general, and non-mutually exclusive, mechanisms have been hypothesized to account for the way in which bacterial invasion and proliferation could be aided by virus-induced alterations to the microenvironment of the airway epithelium. These include virus-induced barrier compromise to the ciliated epithelium, enhanced availability of nutrient cofactors for bacterial growth, and release from innate immune suppression [46]. Whether MPXV infection results in similar opportunities for bacterial superinfection is worth further investigation, highlighting another important aspect of human MPX that bears consideration and study—what is the propensity for MPX bronchopneumonia to be compounded by bacterial superinfection? Given this uncertainty, empiric treatment with antibiotics might prove to be useful for patients with respiratory complications. Other management options—such as pulmonary hygiene, bronchodilation, and ventilator support—are generally limited in low-resource settings, but could be considered when available. 

Temporary and longer-term sequelae may also be considerations for patients recovering from MPX pneumonia. Non-human primates that survived low- to mid-dose experimental respiratory challenge showed foci of necrosis in the lungs, pulmonary fibrosis, and pleural adhesions at the time of post-recovery euthanasia, 21–25 days following infectious challenge [43]. Other necropsy findings show virus dissemination throughout the gastrointestinal tract and antigen deposition in multiple organs (kidney, liver, ovary, etc.). Longitudinal follow-up of monkeypox patients could be useful to determine the frequency, duration, and severity of pulmonary and other possible sequelae of illness (ocular, dermatologic, neurologic, reproductive, etc.).

## 6. Optimization of Supportive Care

**Figure 2:**
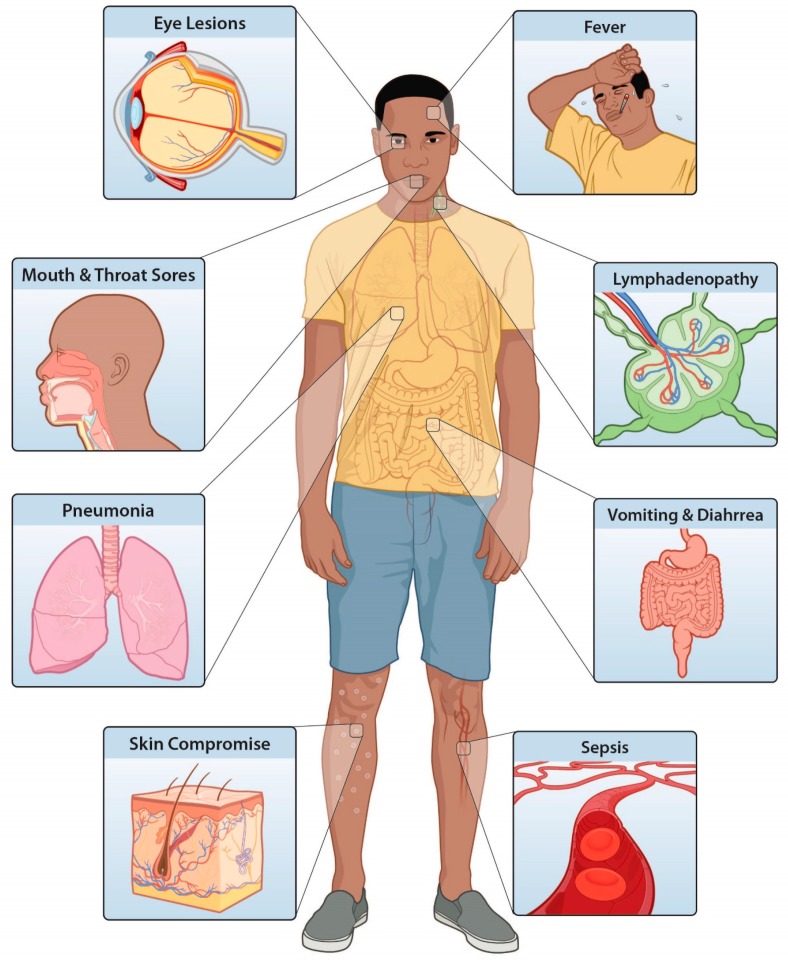
Monkeypox virus infection can have significant impacts on multiple organ systems in the host, including the protective barriers of skin and mucosal surfaces, lymphatics, lungs, and gastrointestinal tract. Skin exfoliation can be significant, and inflammation of the airway and bronchopneumonia can lead to restricted air intake and diminished willingness and/or ability to ingest food and fluids. In rare instances, monkeypox can lead to sepsis. (Illustration: Jennifer Oosthuizen, CDC Division of Communication Services.).

Monkeypox can have significant impacts on multiple organ systems in the host, compromising the protective barriers of skin and mucosal surfaces, provoking a robust focal inflammatory response in the lymphatics, and congestion in the lungs (Figure 2). In instances of heavy rash burden, exfoliation can be significant, subjecting patients to risks from dehydration and protein losses. Serious inflammation and bronchopneumonia can restrict air intake and diminish a patient’s willingness and/or ability to ingest food and fluids. Co-infections (malaria, varicella, HIV) and comorbidities (malnutrition) can also contribute to significant clinical manifestations of illness. An optimal treatment plan for low-resource settings should take into account how likely any of these outcomes are for a given patient [47]. Ideally, this assessment would be based on objective criteria obtained from detailed clinical studies. This would promote the best use of resources to achieve patient recovery while minimizing the chances of onward transmission of the virus. A summary of clinical syndromes associated with monkeypox and potential treatment options for different resource settings is shown in Table 1.

Establishing the practical tools and resource base necessary to provide a minimum standard of supportive care for monkeypox patients, even in low-resource settings, will likely require some degree of investment, including investment in laboratory diagnostics. There is currently an inadequate basis of evidence to answer the question of whether institutional investment in treatment or supportive care would have a sufficient impact on mortality and morbidity to justify the costs. Studies evaluating patient outcomes in relation to treatment intensity, or syndrome-optimized care, would be useful [48]. Alternatives to investing in the resources needed to support a minimum standard of care might include the stockpiling of treatment courses for ocular complications, procurement of vaccine, or provision of personal protective equipment for prevention of healthcare-associated infections. Indeed, many persons with MPX experience only mild to moderate symptoms of illness, but these individuals still constitute a transmission risk until their skin lesions resolve. 

Understanding what proportion of patients—and which patients—are at risk of mortality or significant morbidity will introduce greater efficiency to decision-making with respect to resource allocation. Information valuable to this decision-making process could be generated by focusing on objective, easily measured features of illness or outcomes (e.g., days in hospital, survival, rate of sequelae and complications, pain scores, etc.) Table 2. The relative costs of various interventions and supportive care regimens, accessibility of resources, and patient satisfaction could then be assessed in relation to these outcomes. In cases where national MPX treatment guidelines currently exist, comparison between outcomes optimized for management of specific clinical syndromes versus a standard, regimented approach could be evaluated. 

An additional comment should be made about the possibility of applying countermeasures—developed for the treatment of smallpox in the developed world—to the treatment of monkeypox in Africa. There are no commercially available antiviral drugs for the treatment of monkeypox, but there are (at least) 2 oral investigational compounds (ST-246^®^, SIGA Technologies, Inc., New York, NY, USA; and CMX-001^®^, Chimerix, Inc., Durham, NC, USA) that have shown promise in combatting orthopoxvirus infections [49,50,51]. Both have completed phase 2 safety and efficacy trials in humans, and both drugs can be delivered orally. Neither, however, has been evaluated as a treatment for human monkeypox in a controlled clinical trial. Such trials would require considerable resources to ensure that patients were carefully monitored and able to provide appropriate informed consent prior to study enrollment. Another prerequisite to the clinical application of antivirals would be to understand the degree to which patient outcomes are impacted by the imposition of a basic standard of care (or syndrome-optimized care). Following the demonstration of such, the further benefits of antiviral use could be both qualitatively and quantitatively assessed. 

## 7. Conclusions

The quality and availability of care provided for neglected tropical diseases is recognized as a priority performance indicator for countries striving to achieve disease elimination [52,53]. This objective should also hold true for diseases such as monkeypox, which, though not specifically targeted for elimination, affects almost exclusively persons who do not have access to sophisticated or specialized health care. Establishing evidence-based case management strategies is also a key facet of epidemic preparedness, as outlined in the Integrated Disease Surveillance and Response Technical Guidelines of 2010 [54]. Data from observational studies and animal experiments can inform the approach to improving patient outcomes, but additional clinic-based studies, are also needed to ensure that approaches to care are optimized both for positive outcomes and for efficient utilization of resources.

In recent years, various animal model studies (particularly those in non-human primates) have shed light on the pathophysiology of monkeypox disease, suggesting specific elements of supportive care that may be both practicable and effective at improving patient clinical outcomes. The impetus for these studies was biodefense, emanating from a need to replicate (in an animal model system) severe smallpox-like disease against which to measure the efficacy of medical countermeasures including vaccines and antivirals. These studies have thus far provided clinically useful observations relevant to monkeypox bronchopneumonia and other aspects of the clinical picture of severe illness such as likely indicators of poor outcomes (e.g., percent reduction in body weight at the apex of rash) [43]. However, one obvious question is whether the data are valid for extrapolation to humans. Because the intent is to replicate severe disease, MPXV infection studies using NHPs are rarely performed with low-dose virus challenge, and the dose required for successful infection can vary between different species of host. Both observational and experimental studies suggest that dose influences not only illness severity, but also the length of incubation period and the manifestations of illness in NHPs and in humans [42,55,56]. Respiratory challenge at high doses generally results in compressed incubation periods and respiratory syndromes and as such may have more bearing on the clinical picture of persons affected during MPX outbreaks during periods of significant inter-human transmission (i.e., transmission through respiratory routes). Primary zoonotic, parenteral and mucosal infections may also be prominent and may lead to different clinical tableaus. 

Careful observational studies could shed light on the influence of route of infection and dose on clinical presentation; the rate of occurrence and severity of potential complications and the role of secondary bacterial or viral infections in amplifying the consequences of complications; causes of mortality; and the health consequences of sequelae, both short-term and chronic. Determining early clinical features of illness that are predictive of poor outcomes or mortality could help to optimize the nature of supportive care or treatment, and could have application for the optimization of countermeasure allocation in the event of a monkeypox or smallpox emergency. Monkeypox is an inexact surrogate for smallpox, nonetheless, it is the best extant model for understanding clinical implications of smallpox in a modern context. 

## Figures and Tables

**Table 1:** Monkeypox: Clinical syndromes and possible treatment options.

System Affected/Syndrome	Treatment Objective	Therapeutic Considerations/Clinical Setting		Follow-up/Monitoring
		Developed	Low-Resource	
Respiratory tract	Maintain patent airways, prevent respiratory infection, atelectasis, and respiratory compromise	Suctioning of the nasopharynx and airways, incentive spirometry, chest physiotherapy, bronchodilation, oral/intravenous antibiotics for prophylaxis/treatment, nebulizer treatments, bronchoscopy, noninvasive ventilation (e.g., BiPAP or CPAP) ^1^, intubation/ventilation	Suctioning of the nasopharynx and airways, incentive spirometry, chest physiotherapy, bronchodilation, oral/intravenous antibiotics for prophylaxis/treatment	Respiratory rate, pulse oximetry
Sepsis	Hemodynamic stabilization	Oral/intravenous antibiotics, hemodynamic (e.g., intravenous fluid hydration and vasopressors), supplemental oxygen, corticosteroids, insulin	Oral/intravenous antibiotics, intravenous fluid hydration	Hemodynamic monitoring (e.g., pulse rate, blood pressure)
Gastrointestinal/mouth & throat sores	Minimize mucosal pain and disruption of food intake, promote lesion healing	Oral/topical analgesic medications	Oral/topical analgesic medications	Lesion burden, pain scale, food/fluid intake
Gastrointestinal/vomiting, diarrhea	Minimize gastrointestinal fluid losses	Oral/intravenous antiemetic and antidiarrheal medications, oral/intravenous rehydration	Oral/intravenous antiemetic and antidiarrheal medications, oral/intravenous rehydration	Frequency and volume of emesis and diarrhea, body weight, fluid intake/ouput
Fever	Prevent and treat episodes of fever	Antipyretic medications, external cooling	Antipyretic medications, external cooling	Routine temperature monitoring
Exfoliation, skin compromise	Minimize insensible fluid loss, promote lesion healing	Wash with soap and water or dilute water povidone-iodine solution, moisturized dressings, topical antibiotics (e.g., silver sulfadiazine), surgical debridement, skin grafts	Wash with soap and water or dilute water povidone-iodine solution, moisturized dressings, topical antibiotics (e.g., silver sulfadiazine)	Lesion count/rash burden, body weight, fluid intake/ouput
Superinfection skin	Prevention/treatment of secondary bacterial infections, promote lesion healing	Oral/intravenous antibiotics, incision and drainage, advanced wound management (e.g., negative pressure wound therapy)	Oral/intravenous antibiotics, incision and drainage	Fever, pain/tenderness, erythema, edema, exudate, warmth
Inflammation/lymphadenopathy	Minimize pain and decrease size of lymphadenopathy	Oral/intravenous anti-inflammatory/analgesic medications	Oral/intravenous anti-inflammatory/analgesic medications	Size of lymphadenopathy, pain/tenderness
Ocular infection	Prevent corneal scarring and vision impairment	Ophthalmic antibiotics/antivirals and corticosteroids; slit lamp examination	Ophthalmic antibiotics/antivirals and corticosteroids	Vision testing; repeat examination to assess recrudescence

^1^ BiPAP, bilevel positive airway pressure; CPAP, continuous positive airway pressure.

**Table 2:** Case Management: Suggested Performance Indicators and Clinical Metrics.

Performance Indicator	Clinical Metric	Bench Mark/Target
Reduced mortality	% fatal cases	<5%
Reduced morbidity	% patients provided with supportive care regimen	>50% (good) 1–50% (basic) <1% (inadequate)
Reduced syndrome severity	% patients treated for syndromes/complications (respiratory, epidermal, gastrointestinal, inflammatory)	>50% (good) 1–50% (basic) <1% (inadequate)
Prevention of sequelae	% ocular complications treated with triflouridine	>50% (good) 1–50% (basic) <1% (inadequate)
Prevention of secondary transmission	% patients placed in isolation	>80% (good) 30–80% (basic) <30% (inadequate)

## References

[1] Khodakevich L., Szczeniowski M., Nambu M.D., Jezek Z., Marennikova S., Nakano J., Meier F. (1987). Monkeypox virus in relation to the ecological features surrounding human settlements in Bumba zone, Zaire. Trop. Geogr. Med..

[2] Khodakevich L., Jezek Z., Messinger D. (1988). Monkeypox virus: Ecology and public health significance. Bull. World Health Organ..

[3] McCollum A.M., Nakazawa Y., Ndongala G.M., Pukuta E., Karhemere S., Lushima R.S., llunga B.K., Kabamba J., Wikins K., Gao J. (2015). Human Monkeypox in the Kivus, a Conflict Region of the Democratic Republic of the Congo. Am. J. Trop. Med. Hyg..

[4] Rimoin A.W., Kisalu N., Kebela-Ilunga B., Mukaba T., Wright L.L., Formenty P., Wolfe N.D., Shongo R.L., Tshioko F., Okitolonda E. (2007). Endemic human monkeypox, Democratic Republic of Congo, 2001–2004. Emerg. Infect. Dis..

[5] Rimoin A.W., Mulembakani P.M., Johnston S.C., Lloyd Smith J.O., Kisalu N.K., Kinkela T.L., Blumberg S., Thomassen H.A., Pike B.L., Fair J.N. (2010). Major increase in human monkeypox incidence 30 years after smallpox vaccination campaigns cease in the Democratic Republic of Congo. Proc. Natl. Acad. Sci. USA.

[6] Reynolds M.G., Damon I.K. (2012). Outbreaks of human monkeypox after cessation of smallpox vaccination. Trends Microbiol..

[7] Berthet N., Nakoune E., Whist E., Selekon B., Burguiere A.M., Manuguerra J.C., Gessain A., Kazanji M. (2011). Maculopapular lesions in the Central African Republic. Lancet.

[8] Kalthan E., Dondo-Fongbia J.P., Yambele S., Dieu-Creer L.R., Zepio R., Pamatika C.M. (2016). Twelve cases of monkeypox virus outbreak in Bangassou District (Central African Republic) in December 2015. Bull. Soc. Pathol. Exot..

[9] Khodakevich L., Widy-Wirski R., Arita I., Marennikova S.S., Nakano J., Meunier D. (1985). Monkey pox virus infection in humans in the Central African Republic. Bull. Soc. Pathol. Exot. Fil..

[10] Chen N., Li G., Liszewski M.K., Atkinson J.P., Jahrling P.B., Feng Z., Schriewer J., Buck C., Wang C., Lefkowitz E.J. (2005). Virulence differences between monkeypox virus isolates from West Africa and the Congo basin. Virology.

[11] Likos A.M., Sammons S.A., Olson V.A., Frace A.M., Li Y., Olsen-Rasmussen M., Davidson W., Galloway R., Khristova M.L., Reynolds M.G. (2005). A tale of two clades: Monkeypox viruses. J. Gen. Virol..

[12] Huhn G.D., Bauer A.M., Yorita K., Graham M.B., Sejvar J., Likos A., Damon I.K., Reynolds M.G., Kuehnert M.J. (2005). Clinical characteristics of human monkeypox, and risk factors for severe disease. Clin. Infect. Dis..

[13] Reed K.D., Melski J.W., Graham M.B., Regnery R.L., Sotir M.J., Wegner M.V., Kazmierczak J.J., Stratman E.J., Li Y., Fairley J.A. (2004). The detection of monkeypox in humans in the Western Hemisphere. N. Engl. J. Med..

[14] Jezek Z., Szczeniowski M., Paluku K.M., Mutombo M. (1987). Human monkeypox: Clinical features of 282 patients. J. Infect. Dis..

[15] Jezek Z., Szczeniowski M., Paluku K.M., Mutombo M., Grab B. (1988). Human monkeypox: Confusion with chickenpox. Acta Trop..

[16] Jezek Z., Grab B., Szczeniowski M., Paluku K.M., Mutombo M. (1988). Clinico-epidemiological features of monkeypox patients with an animal or human source of infection. Bull. World Health Organ..

[17] Learned L.A., Reynolds M.G., Wassa D.W., Li Y., Olson V.A., Karem K., Stempora L.L., Braden Z.H., Kline R., Likos A. (2005). Extended Interhuman Transmission of Monkeypox in a Hospital Community in the Republic of the Congo, 2003. Am. J. Trop. Med. Hyg..

[18] Jezek Z., Grab B., Paluku K.M., Szczeniowski M.V. (1988). Human monkeypox: Disease pattern, incidence and attack rates in a rural area of northern Zaire. Trop. Geogr. Med..

[19] Reynolds M.G., Emerson G.L., Pukuta E., Karhemere S., Muyembe J.J., Bikindou A., McCollum A.M., Moses C., Wilkins K., Zhao H. (2013). Detection of human monkeypox in the Republic of the Congo following intensive community education. Am. J. Trop. Med. Hyg..

[20] Bass J., Tack D.M., McCollum A.M., Kabamba J., Pakuta E., Malekani J., Nguete B., Monroe B.P., Doty J.B., Karhemere S. (2013). Enhancing health care worker ability to detect and care for patients with monkeypox in the Democratic Republic of the Congo. Int. Health.

[21] Chapman J.L., Nichols D.K., Martinez M.J., Raymond J.W. (2010). Animal models of orthopoxvirus infection. Vet. Pathol..

[22] Nalca A., Livingston V.A., Garza N.L., Zumbrun E.E., Frick O.M., Chapman J.L., Hartings J.M. (2010). Experimental infection of cynomolgus macaques (*Macaca fascicularis*) with aerosolized monkeypox virus. PLoS ONE.

[23] Zaucha G.M., Jahrling P.B., Geisbert T.W., Swearengen J.R., Hensley L. (2001). The pathology of experimental aerosolized monkeypox virus infection in cynomolgus monkeys (*Macaca fascicularis*). Lab. Investig..

[24] Goff A., Mucker E., Raymond J., Fisher R., Bray M., Hensley L., Paragas J. (2011). Infection of cynomolgus macaques with a recombinant monkeypox virus encoding green fluorescent protein. Arch. Virol..

[25] Stagles M.J., Watson A.A., Boyd J.F., More I.A., McSeveney D. (1985). The histopathology and electron microscopy of a human monkeypox lesion. Trans. R. Soc. Trop. Med. Hyg..

[26] Bayer-Garner I.B. (2005). Monkeypox virus: Histologic, immunohistochemical and electron-microscopic findings. J. Cutan. Pathol..

[27] Patel A.R., Romanelli P., Roberts B., Kirsner R.S. (2009). Herpes simplex virus: A histopathologic study of the depth of herpetic wounds. Int. J. Dermatol..

[28] Graef S., Kurth A., Auw-Haedrich C., Plange N., Kern W.V., Nitsche A., Reinhard T. (2013). Clinicopathological findings in persistent corneal cowpox infection. JAMA Ophthalmol..

[29] Pahlitzsch R., Hammarin A.L., Widell A. (2006). A Case of Facial Cellulitis and Necrotizing Lymphadenitis due to Cowpox Virus Infection. Clin. Infect. Dis..

[30] Patel A.R., Romanelli P., Roberts B., Kirsner R.S. (2007). Treatment of herpes simplex virus infection: Rationale for occlusion. Adv. Skin Wound Care.

[31] Varman K.M., Namias N., Schulman C.I., Pizano L.R. (2014). Acute generalized pustular psoriasis, von Zumbusch type, treated in the burn unit. A review of clinical features and new therapeutics. Burns.

[32] Johnston S.C., Johnson J.C., Stonier S.W., Lin K.L., Kisalu N.K., Hensley L.E., Rimoin A.W. (2015). Cytokine modulation correlates with severity of monkeypox disease in humans. J. Clin. Virol..

[33] Wise L.M., Stuart G.S., Real N.C., Fleming S.B., Mercer A.A. (2014). Orf virus IL-10 accelerates wound healing while limiting inflammation and scarring. Wound Repair Regen..

[34] Zevin A.S., Xie I.Y., Birse K., Arnold K., Romas L., Westmacott G., Novak R.M., McCorrister S., McKinnon L.R., Cohen C.R. (2016). Microbiome Composition and Function Drives Wound-Healing Impairment in the Female Genital Tract. PLoS Pathog..

[35] Bras G. (1952). Observations on the formation of smallpox scars. AMA Arch. Pathol..

[36] Regan T.D., Norton S.A. (2004). The scarring mechanism of smallpox. J. Am. Acad. Dermatol..

[37] Semba R.D. (2003). The ocular complications of smallpox and smallpox immunization. Arch. Ophthalmol..

[38] Hu G., Wang M.J., Miller M.J., Holland G.N., Bruckner D.A., Civen R., Bornstein L.A., Mascola L., Lovett M.A., Mondino B.J. (2004). Ocular vaccinia following exposure to a smallpox vaccinee. Am. J. Ophthalmol..

[39] Lewis F.S., Norton S.A., Bradshaw R.D., Lapa J., Grabenstein J.D. (2006). Analysis of cases reported as generalized vaccinia during the US military smallpox vaccination program, December 2002 to December 2004. J. Am. Acad. Dermatol..

[40] Montgomery J.R., Carroll R.B., McCollum A.M. (2011). Ocular vaccinia: A consequence of unrecognized contact transmission. Mil. Med..

[41] Altmann S., Brandt C.R., Murphy C.J., Patnaikuni R., Takla T., Toomey M., Nesbit B., McIntyre K., Covert J., Dubielzig R. (2011). Evaluation of therapeutic interventions for vaccinia virus keratitis. J. Infect. Dis..

[42] Reynolds M.G., Yorita K.L., Kuehnert M.J., Davidson W.B., Huhn G.D., Holman R.C., Damon I.K. (2006). Clinical manifestations of human monkeypox influenced by route of infection. J. Infect. Dis..

[43] Goff A.J., Chapman J., Foster C., Wlazlowski C., Shamblin J., Lin K., Kreiselmeier N., Mucker E., Paragas J., Lawler J. (2011). A novel respiratory model of infection with monkeypox virus in cynomolgus macaques. J. Virol..

[44] Dyall J., Johnson R.F., Chen D.Y., Huzella L., Ragland D.R., Mollura D.J., Byrum R., Reba R.C., Jennings G., Jahrling P.B. (2011). Evaluation of Monkeypox Disease Progression by Molecular Imaging. J. Infect. Dis..

[45] Bras G. (1952). The morbid anatomy of smallpox. Doc. Med. Geogr. Trop..

[46] McCullers J.A. (2014). The co-pathogenesis of influenza viruses with bacteria in the lung. Nat. Rev. Microbiol..

[47] Nipp R.D., Kelley M.J., Williams C.D., Kamal A.H. (2013). Evolution of the Quality Oncology Practice Initiative supportive care quality measures portfolio and conformance at a Veterans Affairs medical center. J. Oncol. Pract..

[48] Kielstein J.T., Beutel G., Fleig S., Steinhoff J., Meyer T.N., Hafer C., Kuhlmann U., Bramstedt J., Panzer U., Vischedyk M. (2012). Best supportive care and therapeutic plasma exchange with or without eculizumab in Shiga-toxin-producing *E. coli* O104:H4 induced haemolytic-uraemic syndrome: An analysis of the German STEC-HUS registry. Nephrol. Dial. Transplant..

[49] Chittick G., Morrison M., Brundage T., Nichols W.G. (2017). Short-term clinical safety profile of brincidofovir: A favorable benefit-risk proposition in the treatment of smallpox. Antivir. Res..

[50] Jordan R., Chinsangaram J., Bolken T.C., Tyavanagimatt S.R., Tien D., Jones K.F., Frimm A., Corrado M.L., Pickens M., Landis P. (2010). Safety and Pharmacokinetics of the Anti-Orthopoxvirus Compound ST-246(R) following Repeat Oral Dosing in Healthy Adult Subjects. Antimicrob. Agents Chemother..

[51] Jordan R., Leeds J.M., Tyavanagimatt S., Hruby D.E. (2010). Development of ST-246(R) for Treatment of Poxvirus Infections. Viruses.

[52] Lonnroth K., Castro K.G., Chakaya J.M., Chauhan L.S., Floyd K., Glaziou P., Raviglione M.C. (2010). Tuberculosis control and elimination 2010–50: Cure, care, and social development. Lancet.

[53] World Health Organization (2000). Leprosy Elimination Monitoring (LEM); Guidelines for Monitors.

[54] Kasolo F., Raungou J., World Health Organization Regional Office of Africa Disease Prevention and Control Custer, US Centers for Disease Control and Prevention Center for Global Health (2010). Technical Guidelines for Integrated Disease Surveillance and Response in the African Region.

[55] Mucker E.M., Chapman J., Huzella L.M., Huggins J.W., Shamblin J., Robinson C.G., Hensley L.E. (2015). Susceptibility of Marmosets (*Callithrix jacchus*) to Monkeypox Virus: A Low Dose Prospective Model for Monkeypox and Smallpox Disease. PLoS ONE.

[56] Tree J.A., Hall G., Pearson G., Rayner E., Graham V.A., Steeds K., Bewley K.R., Hatch G.J., Dennis M., Taylor I. (2015). Sequence of pathogenic events in cynomolgus macaques infected with aerosolized monkeypox virus. J. Virol..

